# Dynamic 3D On-Chip BBB Model Design, Development, and Applications in Neurological Diseases

**DOI:** 10.3390/cells10113183

**Published:** 2021-11-15

**Authors:** Xingchi Chen, Chang Liu, Laureana Muok, Changchun Zeng, Yan Li

**Affiliations:** 1 Department of Chemical and Biomedical Engineering, FAMU-FSU College of Engineering, Florida State University, Tallahassee, FL 32310, USA; xc19a@my.fsu.edu (X.C.); cl20ev@my.fsu.edu (C.L.); laureana1.muok@famu.edu (L.M.); 2 The High-Performance Materials Institute, Florida State University, Tallahassee, FL 32310, USA; 3 Department of Industrial and Manufacturing Engineering, FAMU-FSU College of Engineering, Florida State University, Tallahassee, FL 32310, USA; zeng@eng.famu.fsu.edu

**Keywords:** blood–brain barrier, human pluripotent stem cells, microfluidics, three-dimensional model, neurological disorders

## Abstract

The blood–brain barrier (BBB) is a vital structure for maintaining homeostasis between the blood and the brain in the central nervous system (CNS). Biomolecule exchange, ion balance, nutrition delivery, and toxic molecule prevention rely on the normal function of the BBB. The dysfunction and the dysregulation of the BBB leads to the progression of neurological disorders and neurodegeneration. Therefore, in vitro BBB models can facilitate the investigation for proper therapies. As the demand increases, it is urgent to develop a more efficient and more physiologically relevant BBB model. In this review, the development of the microfluidics platform for the applications in neuroscience is summarized. This article focuses on the characterizations of in vitro BBB models derived from human stem cells and discusses the development of various types of in vitro models. The microfluidics-based system and BBB-on-chip models should provide a better platform for high-throughput drug-screening and targeted delivery.

## 1. Introduction

Investigation of the function and the original system of the nervous network helps to acquire an understanding of nervous system disorders and medical treatments [1]. The central nervous system (CNS) consists of the brain and the spinal cord, and controls most body and mind function. The brain is the core of the CNS, which can generate thoughts, interact with the external environment, and direct movements. Therefore, impairment of the CNS can lead to severe problems in maintaining normal human life [2]. Neurological diseases become common due to aging and societal improvements. Alzheimer’s Disease (AD), for example, was diagnosed in 2.4 million people over the age 65 in 2017 and it is expected to double in over next three decades [3,4,5]. Besides AD, Parkinson’s disease (PD), multiple sclerosis, stroke, and traumatic brain injuries, which impair neural tissues, are also attracting more attention. Despite the increasing demand for treatment of CNS diseases, the fragile CNS structure makes traditional surgeries difficult. Therefore, the need for therapies to treat CNS diseases requires the development of new therapy technologies. Despite such emerging demands for the treatment of CNS diseases, only 7% of CNS drugs in clinical development reach the marketplace, compared to the 12% average across all therapeutic areas in 2007, and ten years later only 113 drugs have been proved by the Food and Drug Administration (FDA) compared with the 2313 oncology therapies [6,7,8].

**Figure 1:**
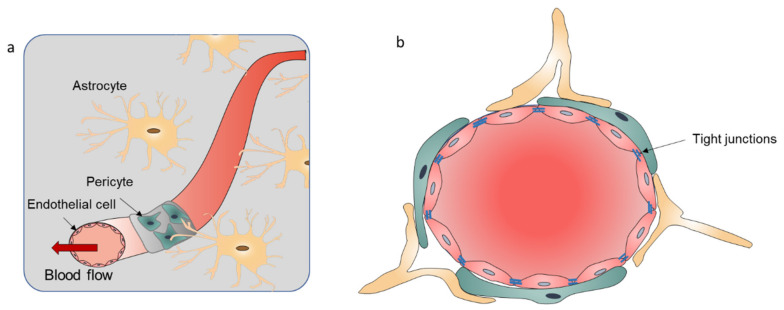
(**a**) Schematic illustration of the BBB components. The BBB is consisted by the endothelial cell monolayer with tight junctions, pericytes wrapping around a blood vessel, and the astrocyte touching the blood vessel with its end foot. (**b**) The cross section of the BBB.

This low development rate of the trials for CNS diseases is attributed to an important brain structure known as the blood–brain barrier (BBB). The BBB is a highly functionalized border which ensures the separation between blood and the CNS. This structure maintains normal physiological function of brain cells and cerebral homeostasis. The BBB has a three-dimensional (3D) network of astrocytes that communicate with endothelial cells (ECs) and pericytes, which dynamically modulates and controls the balance of molecules and ions between the blood and the brain cells [9,10,11]. The core component of the BBB is the ECs, which are arranged as the vascular tubular vessel structure through tight junctions (TJs). The integrity of the BBB is maintained by the pericytes, which support the glial cells (astrocytes and microglia), basement membranes, and the extracellular matrix (ECM) (Figure 1) [12,13,14]. The connection between astrocytes and vascular cells controls the influx of the water. The transporter proteins and TJs of the BBB interfaces regulate the passage of nutrients, and protect the brain from toxins and pathogens due to the blood circulation in the CNS [15,16]. Dysfunction of the BBB can change the barrier permeability, and influx and efflux between the blood and the CNS, leading to the infiltration of toxins or immune cells and the development of neurological disease [17,18]. The complex architecture of the BBB, the tight barrier integrity, and the availability of specific molecules that can pass BBB lead to a low success rate in the development of therapeutics for treating CNS diseases [19]. Targeted drug-oriented delivery and release is the best way to treat these CNS diseases. Developing relevant models to accurately reproduce the structure of BBB and monitor the behavior of complex interactions with brain tissue helps to understand the mechanism of neurological disease development and identify new treatment methods [20]. BBB provides by far the largest surface area for molecule exchange and in the adult human it is about 12–18 m^2^ in surface area [21]. After crossing the capillaries (less than 25 µm), the diffusion distance of the drugs and nutrition to neurons and glial cells is short. Therefore, developing a drug that can cross the BBB is the main strategy for the CNS disease therapies [22]. There are many approved drugs entering the market for treating BBB-related diseases every year, but the efficacy of the therapies is less than ideal. A proposed solution is the use of drug-targeted delivery to acquire higher efficiency. The key to this strategy is to investigate the transport mechanism through ECs in a good BBB model to help researchers understand and design the proper modifications of the drugs for treating brain dysfunctions. 

Traditionally, there are three types of BBB models: parallel artificial membrane permeability assay (PAMPA) models, cell-based transwell models, and animal models. The PAMPA and the transwell models can mimic 2D interactions in static culture systems. Lacking the physiological structure and functionalities, the results cannot predict the behaviors of the BBB for clinical outcomes. The recent advancement in the BBB-related research has applied the microfluidics and fabricated dynamic in vitro BBB models with integrated sensors to recapitulate the blood and brain microenvironment [23,24]. These BBB-on-chip models are also called microfluidic BBB (μBBB), mimicking the micro-physiological system to meet the basic function requirements of BBB in vitro. The highly controlled model devices allow people to fabricate a relatively similar microenvironment of BBB along with the blood and different cell layers using coculture systems. Existing BBB models can reconstruct the tight junctional EC barrier in different culture systems, including monoculture of brain ECs [25] and coculture of ECs with astrocytes/pericytes in 2D and 3D microenvironments [26,27]. Moreover, these BBB models can be combined with high-resolution imaging and drug-screening platforms, enabling the monitoring of intercellular and extracellular behaviors. One advanced development of the in vitro BBB models is to introduce shear stress on ECs, known as dynamic in vitro BBB systems [28]. The mechanisms of BBB in brain function can be investigated through these in vitro BBB models, which can provide the proper strategies for drug delivery. Thus, BBB-on-chip models possess a great potential for a wide range of applications, including CNS disease modeling, high-throughput screening (HTS) of new therapeutics, and neurotoxicity testing. 

In this review, advanced in vitro BBB model development is discussed. First, the biological properties of the BBB, including the architectures, function, cell types, and methods for characterizations are summarized. Then, various BBB in vitro models are presented, including traditional models and the dynamic 3D models. Next, various designs of the in vitro BBB devices are listed with various selected materials. The applications of the in vitro BBB models are shown with the advanced concepts. Finally, the review is concluded with perspectives for future BBB model research.

## 2. Current Design of BBB Models

### 2.1. Introduction of the BBB

**Figure 2:**
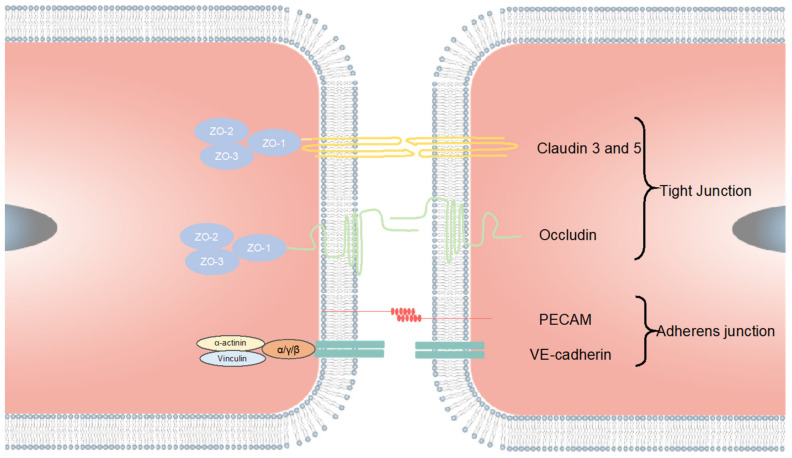
Tight junction formation of the ECs. The TJs are mainly combined by claudin 3, claudin5, occludin, and other possible claudins. The PECAM and VE-cadherin form the adherens junction. The tight junctions between ECs prevent molecules from easily crossing the EC layer. The claudins and the occludin are connected with the scaffolding proteins ZO-1, ZO-3, and ZO-3, which are linked to the myosin/actin cytoskeleton.

**Figure 3:**
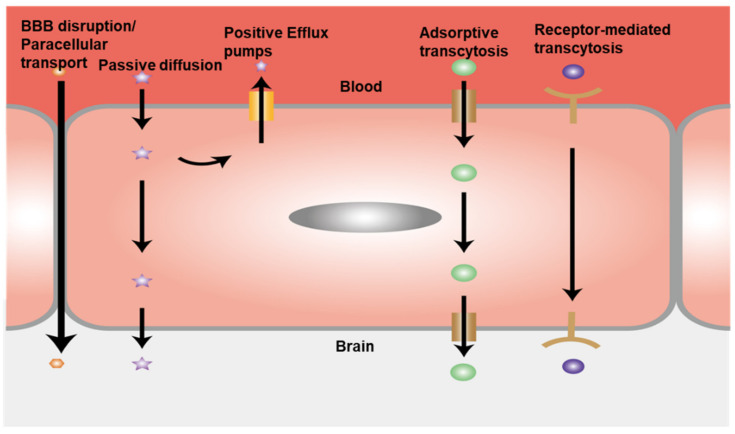
Schematic illustration of BBB transport pathways. The activity of efflux transport proteins plays important roles in the function of the BBB. The complex TJ structure of the BBB forces most molecular trafficking to a transcellular route across the BBB. Transport proteins can actively carry essential biomolecules across the BBB. Receptor-mediated transcytosis is another positive transport route, where certain peptides and proteins, such as insulin and transferrin, are selectively transferred. The large hydrophilic molecules can be transported by the adsorptive-mediated endocytosis route.

The BBB is one of the CNS barriers between the brain and the blood with a dynamic multifunctional layer formed by the wall of cerebral capillary endothelial cells due to tight junctions (TJs) [19,29,30]. There are three types of proteins that hold the functionalities of the tight junctions: tight junction proteins, adherens junction proteins, and scaffolding proteins. (Figure 2). These transmembrane proteins include protein crumbs homologue 3 (CRB3); MARVEL domain proteins such as occludin, tricellulin and MARVEL domain-containing protein 3 (MARVELD3); claudins; blood vessel epicardial substance (BVES); and junctional adhesion molecules (JAMs), as well as the adaptor proteins, cytoskeletal linkers, Zonula occludens (ZO) proteins (ZO1, ZO2 and ZO3); and cingulin; partitioning defective 3 (PAR3). The nectins and the VE-cadherin form the TJs which work with their main cytosolic interaction partners for cell–cell interactions [31,32]. These proteins control the pathways by regulating the balance of ions and the crossing of biomolecules. Due to the selective property of the BBB, many biomolecules that need to enter the brain go through transcellular route, causing the failure of prospective drugs for treating CNS diseases due to the ineffective transport. The small lipophilic molecules such as the exosomes and liposomes have been investigated as the new drug vehicles for the CNS disease treatment, because of their ability to cross BBB without restrictions [31]. The tight junctional EC layer is the vital component of the BBB structure, while the other components such as pericytes, astrocytes, microglia, and the ECM are all indispensable for maintaining the BBB function [33,34,35]. The normal function of astrocytes is to maintain barrier stability and prevent BBB disruption. Healthy astrocytes have branches around the cell bodies and express AQP4 at the end feet which contacts the vascular cells [36,37]. Otherwise, the abnormal astrocytes contribute to the disruption of barrier integrity, leading to increased permeability and thus neurodegeneration, ischemia, and infection [38]. Besides the astrocyte dysfunction, any deactivations of other components can lead to BBB-related diseases. The penetration of the toxic materials, blood cells, and other components through the vessel to the CNS due to increased permeability leads to the dysregulation of the influx and efflux through BBB (Figure 3). The emergence of neurological diseases including AD, PD, and multiple sclerosis are all related to BBB damage. Therefore, it is necessary to establish platforms for accurately recapitulating the BBB function and its interactions with brain tissue. In this way, more precise and intuitive approaches can be developed to investigate neurological disease progression, perform drug-screening, and formulate drug delivery strategies.

### 2.2. Computation Models

Due to advancements in the field of computer science, the BBB can be modeled by a computer. Most computational models are applied to BBB penetration. Data mining methods such as multiple linear regression [39], partial least square regression, excursive partitioning [40], neural network [41], and support vector machine (SVM) [42] have been widely employed in BBB penetration models. In addition, the models can be used to predict the BBB permeability which are associated is with the physical and chemical properties of the biomolecules and the BBB, such as the topology of surface, wettability, hydrogen bonds, and the receptors. One type of the models, known as classification models, is applied to distinguish between the biomolecules capable of crossing the BBB and those restricted to the periphery [43]. This model has already compiled more than 1500 drugs with the published data [44]. For example, Zhang used SVM regression combined with the genetic algorithm to optimize kernel parameters. According to their model, the carboxylic acid group, polar surface area (PSA)/hydrogen-bonding ability, lipophilicity, and molecular charge play important roles in BBB penetration [42]. However, most results obtained using computation simulation need to be verified by in vivo experiments [45,46,47].

### 2.3. In Vivo Models

Due to the complexity and the high cost of in vivo experiments, there have been few in vivo BBB models developed in recent decades. However, in the absence of advanced in vitro models, in vivo models are still important for examining drug efficiency to treat CNS diseases. These models can closely mimic the physiological environment of the human BBB. Modeling results can be used for predicting the outcomes of new drugs and therapy efficacy [48]. The most common ways to construct in vivo models include intravenous injection, brain perfusion, positron emission tomography, and microdialysis sampling [49]. The nature-mimicking system could generate reliable data for human CNS therapies. Li et al. report a systematic model using zebrafish, which is good for setting a timely, reproducible model for BBB permeability study [50]. Liu et al. used different sizes of polyethylene glycosylated silica nanoparticles for investigating the transport efficiency of silica nanoparticles (the bigger diameter the lower permeability of the NPs). This work demonstrates the potential application for drug delivery across BBB [51]. However, in vivo animal BBB models cannot be straightforwardly used for predicting and establishing a dependable relationship with the actual human CNS activities. Although the same experimental conditions have been attempted, there still exist large animal-to-animal variations, and discrepancy from the human BBB function and microenvironment. Using the in vivo models also suffers from increased cost and the labor, and low efficiency for high-throughput screening [52].

### 2.4. In Vitro Models

In vitro BBB models are highly efficient models. It is easy to construct the blood–brain barrier structure and operate the model in experiments. There are many methods to fabricate diversified in vitro BBB culture systems, which are classified as static and dynamic models (Table 1). The static models are usually the traditional mono- and multi-cell culture in transwells, brain slice culture, and PAMPA. The static models are easy to control and observe. As for the dynamic models, the dynamic fiber-based BBB (DIV-BBB) model was designed in 2006. With the development of the microfluidic technology, µBBB models have been developed recently.

#### 2.4.1. Static In Vitro BBB Models

Static in vitro BBB models have been used for decades and generated good research results for understanding the basic activities of the BBB. At first, the transwell was used to provide the membrane for mimicking the BBB structure. The existence of the membrane-integrated cell culture system makes it easy to provide two different environments and distinguish the blood side and the brain side. The mono- (focusing on barrier properties) and multi-cell culture (has cell–cell interactions) systems have both been investigated. The transwell model has the advantages for testing drug delivery efficiency and permeability through the EC layer. As mentioned earlier, the EC layer is the key barrier component for the control of biomolecules crossing the barrier. There are three different types of ECs commonly used in transwell models, the human umbilical vein endothelial cells (HUVEC), human cerebral microvascular endothelial cells (hCMEC), and primary human vascular endothelial cells [66]. Recently, human pluripotent stem cell (hPSC)-derived brain microvascular endothelial cells have emerged as promising EC sources for in vitro BBB models [67,68,69]. The static BBB model is easy to build and widely used for modeling the biomolecule transport through the BBB in vitro. For example, Wainwright et al. used two different cell models, the mouse bEnd.3 cells and primary porcine brain endothelial cells (PBECs), to investigate the transport of primary coenzyme Q_10_ (CoQ_10_) which can treat mitochondrial respiratory chain disorders. The transport mechanism of CoQ_10_ was investigated in normal and pathophysiological models. It is the first time that a model of transcytosis of lipoprotein associated CoQ_10_ has been established, and discovered the mechanisms of regulating the CoQ_10_ uptake and efflux between the two sides of the BBB [70]. Besides the traditional transwell, the hydrogel culture system was introduced to fabricate the 3D BBB model to mimic in vivo microenvironment. Augustine et al. used gelatin methacryloyl (GelMA) modified transwell to build a 3D culture system for investigating the anti-metastatic agent against metastatic breast cancer [71]. Astrocytes were mixed with the GelMA and then the system was crosslinked by UV exposure. ECs were then seeded on the gel to form the TJ barrier followed by the cancer cell seeding. Using this model, the anti-metastatic agent cisplatin was shown to depress cancer cell migration across the BBB.

Besides astrocytes, brain pericytes have been included in the in vitro BBB models. For example, a multi-cell culture model was designed by Stebbins et al. to demonstrate that pericytes play important roles in the formation and physiological function of the BBB [59]. Therefore, brain pericyte-like cells, differentiated from hPSC-derived neural crest stem cells (NCSCs), were cocultured with endothelial cells, neurons, and astrocytes. This integrated culture system rebuilt an isogenic human BBB model. However, shear stress was not applied in this model, which may lead to inaccurate results. Hence, the static BBB models need to be integrated with microfluidic devices to reveal the mechanism of BBB regulation in the presence of shear stress which can better develop clinical treatments for neurological diseases.

#### 2.4.2. Dynamic In Vitro BBB Models

Due to lack of shear stress in static models, dynamic BBB models have attracted increasing attention over the last decade. At first, the humanized DIV-BBB model was established, in which the ECs were cultured in the capillaries where the physiologic levels of shear stress generated by intraluminal flow can be applied [72]. In addition, the capillaries are surrounded with other chambers which provide the simulation of different brain regions. This 3D dynamic in vitro BBB system can generate more reliable data. For example, Cucullo et al. used this model to investigate brain penetration of anti-epileptic drugs [72]. Then, they revised their DIV-model with transmural microholes to allow the transport of the immune cells and recapitulate the original physiological characteristics of the BBB. These microholes do not inhibit the generation of the TJ barrier. The permeability level of sucrose, phenytoin, and diazepam was successfully investigated and the existence of the microholes also enables the study of immune cell migration cross the BBB [73].

However, this DIV-BBB model is not widely used due to several limitations. For example, it requires longer culture time to reach the maximum value of the transepithelial/transendothelial electrical resistance (TEER). In addition, all the BBB areas are in one integrated chamber which makes it difficult to observe cellular behaviors. Moreover, the wall of the capillaries is much thicker than the porous membranes, the key components in the µBBB models. The thickness reduces the contact of ECs with the pericytes, astrocytes, or neurons [74].

To overcome the disadvantages of static BBB models and DIV-BBB models, the BBB-on-chip models were designed with the development of microfluidic technologies. The BBB-on-chip models consider the effects of the blood flow in the neural tissue and can be used for the specific screening of the transporting molecules. Prabhakarpandian et al. developed a simple microfluidic vasculature model of the BBB with a horizontal-aligned structure [75]. Partyka et al. showed a 3D model of the BBB composed of two horizontal channels and a hydrogel reservoir at the center of the two channels [63]. However, many dynamic BBB models are based on the 2D systems which ignore the structure of 3D blood vessels. The use of tubular 3D structures can provide better contact of the BBB cells with their environment, i.e., neural tissues and glia cells can have a greater interaction with the EC barrier. Although it is difficult to establish a stable, complete 3D structure in vitro, there have been several attempts to develop an in vitro 3D BBB model using artificial channels. For example, Kim et al. developed a 3D in vitro brain microvasculature system embedded within the bulk of a collagen matrix [76]. They used the 40 kDa fluorescein isothiocyanate-dextran for characterizing the permeability through the microvessel models. In addition, the recovery behaviors of brain disruption in this model were also examined.

## 3. Principles of Microfluidic Device Design

A perfect in vitro BBB model needs to recapitulate all the features of the BBB in vivo, such as the structure of ECs, cell–cell interactions, controlled flow (in particular shear stress on ECs), and a molecular transportable basal membrane (BM). Most μBBB models use the porous membrane segmentation to form sandwich structures in the chip that are similar to those used in transwell systems. ECs and the other cells are cultured on different sides of the membrane which provide different microenvironment acting similar to a neural chamber next to a vascular chamber. The coculture models indeed overcome the limitations of conventional 2D cultures, including altered cell morphologies and gene expression. To maintain the function of the brain tissues, cell–cell interactions have vital roles, such as tissue regeneration and repair. Therefore, the coculture approach provides indispensable properties in future BBB models, but still faces the challenges for recapitulating the BBB in vitro. The choice of materials for the basal membrane is one of the challenges. The BM is involved in several process including cell differentiation, homeostasis, tissue maintenance, and cell structural support. Ideally, an artificial BM should be made of biocompatible materials and have a thickness of ~100 nm [77]. To better mimic the BBB in microfluidic systems, different designs, culture strategies, and materials have been investigated and validated. The reported well-designed microfluidic BBB models are summarized in Table 2.

### 3.1. Chip Materials

The most widely used chip material is the polydimethylsiloxane (PDMS). PDMS is cost-effective, easily shaped, and biocompatible, making it a good choice for fabricating biomedical devices [93]. PDMS is a polymeric organosilicon compound that is optically transparent, non-toxic, non-flammable, and gas- and water-permeable. Devices made by PDMS have good transparence for observation and photography of fluid flow and cell behaviors. Fabrication of PDMS channels is involved with the molds that are etched to produce different well-designed patterns. Then PDMS is peeled off to obtain the channels with or without patterns. PDMS, glass, or other silica material can be easily integrated with the culture systems after plasma treatment [47,94]. In addition, PDMS is flexible and can be made into the channels with different 3D structures. By silanized modification, the wettability of PDMS surface can be easily altered according to the ideal experimental conditions. Although the PDMS seems to be the best material for the microfluidic device, there are still several limitations. The modification process is necessary for the BBB model design because of the hydrophobic surface of PDMS. It makes the hydrophobic molecules easily absorbed with low cell adherence. The coating step may introduce molecules that are not wanted in the experiments or other hazardous substances. Furthermore, some non-crosslinked monomers may leak from the PDMS into culture medium, leading to unexpected cell behaviors [95,96]. Therefore, other microfluidic chips were designed to overcome these limitations. For example, poly methyl methylacrylate (PMMA) [97], one of the transparent thermoplastics, is developed as the substitute to the PDMS. This material has better light transmission, higher chemical stability, better compatibility with organic solvents and biocompatibility than the PDMS. However, thermoplastic materials may not be easily processed into complex micro- and nanostructures.

### 3.2. Microfluidic Device Structure Design

**Figure 4:**
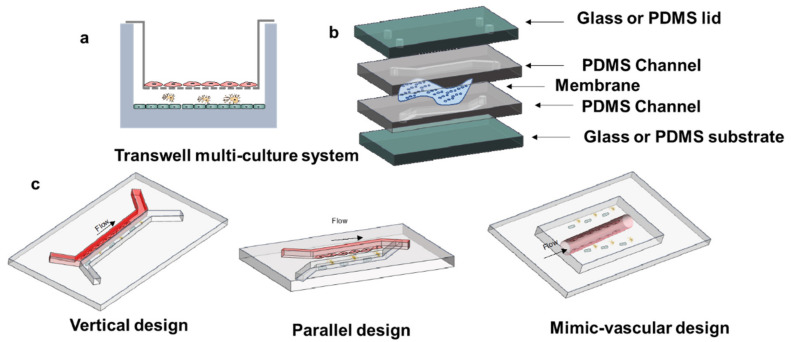
The BBB model development and BBB-on-chip design. (**a**) multi-culture system in the transwell. (**b**) Basic design thought of microfluidic BBB model. (**c**) Different BBB-on-Chip designs.

Initially, the design of the BBB-on-chip devices focuses on how to introduce fluid flow fluid in the models (Figure 4a). Therefore, the classic transwell system was placed in the microfluidic devices to generate a vertical structure similar to a sandwich (Figure 4b). The different channels were separated by the porous membrane. In general, the ECs are cultured in the upper channel to form the barrier and the other cells are cultured in the lower channel to mimic cell–cell interactions. The vertical models have been used widely in the dynamic BBB models due to the convenience of fabrication. However, this kind of model has some inevitable drawbacks. Due to the vertical structure, the effect of gravity makes the cells settle toward the channel bottom. This settling effect results in less interactions between the EC barrier and the other BBB cells. The channel height also influences the contact between different types of cells. In addition, the vertical structure makes it difficult to observe cell behaviors or other movements in the channels due to the presence of membrane. The cell attachment and the biomolecular transport cannot be well monitored in real time. The transparency of the films should be optimized, or the material needs to be replaced for a better observation. The polyethylene terephthalate (PET) and the poly (tetrafluoroethylene) (PTFE) membranes would be good substitutes.

The parallel channels have been designed recently for better observation compared to vertical models. The membrane of parallel models is typically replaced by PDMS channels during channel fabrication. For example, the micropillars with a gap of 3 μm were used to separate the blood from the brain-mimicking chamber, then the tumor cells were seeded to investigate cancer evasion [46]. The integrity of this model makes the device fabrication easy and avoids the use of an extra membrane part. In addition, this kind of design improves the observation of cell behaviors with high-resolution images. Nonetheless, the PDMS membrane has much bigger pores and provides thicker barrier than the nature BM. Due to the flexibility of the PDMS, it is difficult to make a stable thin membrane structure by photolithography. Moreover, the fabrication method has drawback in constructing a tubular vessel-like structure. The shear stress in the tubular structure can be designed under the similar conditions of the brain. This tubular geometry design usually uses porous microneedles, which can be fabricated with different diameters and pores. These 3D microvascular tubes with ECs were further cocultured with astrocytes or pericytes imbedded in a collagen matrix [26].

The BBB-on-chip with the use of ECM gels has been developed as a better model recently. Collagen gel is a commonly used material to form ECM barriers in BBB devices. The ECM hydrogels are frequently used as the brain side materials. The ECM gel-based barriers are found to have similar properties to the basal membrane in the in vivo BBB. As mentioned earlier, the hydrogels become widely used in recent research for building 3D BBB models. Hydrogels can be well-designed for any type of cells and provide a 3D environment for the coculture system. By changing physical or chemical properties, the characteristics of hydrogels can be significantly modified, such as biocompatibility, cytotoxicity, permeability of molecules, degradation rate, etc. For example, the porosity of the hydrogels varies due to different material type, composition of polymers, and the degree of crosslinking [98]. Side groups or branched chains on the polymer can influence cell adhesion. In addition, biomolecules such as peptides and proteins can be conjugated with the chains of the polymers for controlled interactions with cells. Furthermore, the stiffness of the hydrogels can be controlled by different treatments, with the range of less than 1 kPa for mimicking brain environment. Usually, the hydrogels provide a 3D culture environment as scaffolds in cell culture models [99]. There is no need to use a membrane for constructing the blood vessels and brain environment. The bulk hydrogels with good porosity can provide the required permeability of molecules and support the ECs to form the barrier. In general, there are four types of fabrication methods of hydrogels used in microfluidics—soft lithography, extrusion-based bioprinting, light-based 3D bioprinting and laser-based photopatterning [100]. The hydrogels can be loaded with astrocytes, pericytes, and neurons for the coculture system. The EC layers can be generated on the surface of the gels [84]. The hydrogels-based BBB models have been used to investigate cell spreading, tumor penetration, and angiogenesis.

### 3.3. Porous Membrane

As mentioned before, the membranes are usually used to separate the luminal and abluminal layers. The membranes provide barriers for co-culturing endothelial cells and other BBB-associated cells, and make permeability testing possible. Polycarbonate (PC), polyester (PE), polyethylene terephthalate, and polytetrafluoroethylene have been used to fabricate porous membranes in the in vitro BBB models [79].

### 3.4. Cell Source for In Vitro BBB Models

Astrocytes and pericytes play important roles in the reconstruction of the BBB, but brain microvascular ECs are the main cell type that maintains the physical barrier between the blood and the brain. The primary ECs have many favorable properties for building the BBB models. These cells have high gene expression and provide the better BBB phenotype. However, the high expense, the difficulty of cell isolation, and the availability of human cell source require the alternative cell sources for constructing in vitro BBB models [80].

Immortalized cell lines have been used as one of the primary EC alternatives. The cells can be easily purified and passaged over long periods. Human brain microvascular endothelial cells (hCMEC/D3 and hBMECs), and HUVECs are the most frequently used EC sources. HUVEC line can form barriers with the desired permeability in the in vitro BBB models. hCMEC/D3 line exhibits high gene expression as well as proteins and receptors, and the bBMEC line can form more stable dynamic barrier. Therefore, hCMEC/D3 and bBMEC lines are reasonable cell sources for BBB modeling [84].

Using the immortalized cell lines for the BBB models may make TJs less stable. In addition, it is difficult to obtain sufficient number of primary cells for drug-screening and disease model development. Therefore, the ECs (as well as astrocytes, brain pericytes, and neuronal cells) derived from hPSCs are attractive cell sources for constructing the in vitro BBB models. The hPSC-derived cells can be used in the coculture systems with the homologous differentiated cell populations. The hPSC-ECs show the presence of many TJ proteins and endothelial transporters when co-culturing with astrocytes. In addition, the EC barrier has similar characteristics to the natural BBB. In several reports, the hPSC-ECs were used in establishing the in vitro BBB models to recapitulate the in vivo BBB physiology [101,102]. The summary for hPSC-derived BBB models and hPSC-derived brain pericytes has been reviewed in our previously published articles [103,104].

### 3.5. Incorporation of Shear Stress

The flow applied in the microfluidic devices can provide the similar shear stress condition to the natural BBB. Under the influence of the fluid flow, the behaviors of the cells are different from the static conditions [105]. With the exposure of cells to a laminar flow-induced shear stress, the EC barrier shows high expression of the TJ proteins and more integrated barrier properties than other in vitro models in the absence of shear stress. Physiological shear stresses can range from 4–30 dyne/cm^2^ to 1–4 dyne/cm^2^ in the venous circulation. There are a series of methods to stimulate shear stress profiles around the EC barrier in the tubular structure of the microfluidic devices [10,106,107].

## 4. Characterizations to Examine the Model Integrity

### 4.1. Transepithelial/Endothelial Electrical Resistance Measurement

The TJs of the brain endothelium restricts the movement of small ions (such as Na^+^ and Cl^−^), resulting in a measurable electrical resistance, known as TEER. Typically, there are two electrodes placed on two sides of the membrane for measuring the TEER value. In general, the TEER value of the in vitro BBB models should be close to 1800–2000 Ω·cm^−2^, which is in the range of the natural TEER values of the in vivo BBB. However, most of the models have the values less than the standard and 150–200 Ω·cm^−2^ is the lowest acceptable TEER value. In further research, primary cell-based BBB models have the measured TEER values ranging in 600–1800 Ω·cm^2^. In addition, hPSC-derived ECs have been applied to produce physiological TEER values in the range from 4000 to 5000 Ω·cm^2^ in vitro [108]. These results indicate that the cell source is one of the most relevant factors for obtaining a desirable TEER value. Although TEER value is a gold standard for measuring the integrity of in vitro BBB models, some in vitro BBB models cannot allow the TEER measurement [106]. The ECM gel-based BBB models cannot use the TEER value to determine the tightness of EC layer due to the inability to provide TEER measurement. In addition, the endothelial layer is easy to be disrupted when introducing the electrode into the “blood” channel of the ECM gel device.

### 4.2. Tight Junction Markers

In the blood–brain barrier, cells have tight junctions and adhesion molecules at the junctions between endothelial cells to maintain the integrity of the barrier. Immunofluorescence or Western blots can be used to measure the expression of specific markers TJs are formed by occludin, claudins, junctional adhesion molecules (JAMs), and ZO-1, 2, and 3 [109]. Occulin is the most indispensable protein and expressed commonly in TJs. Therefore, occulin is a reliable immunochemical marker [110]. In addition, ZO-1 is the vital part for forming tight junction. Without ZO-1, TJ cannot be assembled [111]. One of the membrane transporters, P-glycoprotein efflux pump regulates the penetration of hydrophobic molecules. Expression of P-glycoprotein is also used to evaluate BBB characteristics in microfluidic-based in vitro BBB models [112].

### 4.3. Permeability

Another important property of the BBB is the penetration control of the biomolecules crossed from the blood through EC layers to the brain area. Ions, lipophilic molecules, polar molecules all need the specific transporters or receptors to cross the BBB [113]. Generally, physicochemical properties such as size and polarity affect the permeability of the biomolecule through the BBB. Dextran is a molecule with a high molecular weight, so the permeability results from dextran of different molecular weights may not fully reflect the integrity of the BBB compared to small molecules with molecular weight under 900 Da [74]. Therefore, the dyes under 900 Da can be used as the label for the permeability experiments [114].

## 5. Applications of In Vitro BBB Models in Neurological Diseases

**Figure 5:**
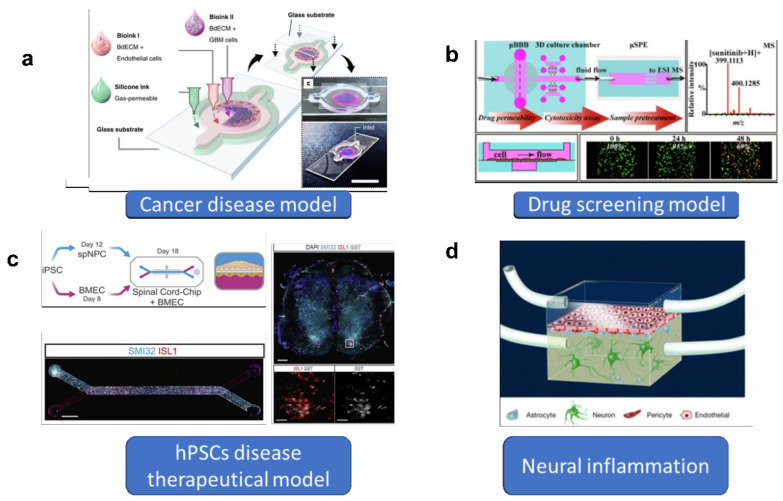
Applications for the BBB-on-chip model. (**a**) Microfluidics for tumor cell filtration investigation. Reproduced with permission from [115] Copyright (**b**) High-throughput drug-screening microfluidics BBB model. Reproduced with permission from [116] Copyright, (**c**) microfluidic hPSC-derived cells for therapeutical strategies. Reproduced with permission from [117] Copyright, (**d**) Microfluidic devices to investigate neuroinflammation.

The BBB-on-chip models can provide more accurate microenvironments by accounting for brain-mimicking conditions, such as the presence of shear stress (Figure 5). These models can be applied to the research and development activities of various types of neurological diseases, such as brain tumors, AD, PD, and multiple sclerosis, for disease modeling, drug testing, and neuroinflammation modulation.

### 5.1. Brain Tumor Research

For brain tumors, lack of effective drug treatments and limited understanding of disease mechanisms are the main reasons for poor treatment effects and high tumor recurrence rates after surgical intervention, radiotherapy, and chemotherapy. BBB models have been used to investigate the interactions between vascular glioma initiating cells, which play a vital role in the invasion of brain tumor cells [118]. In addition, it is possible to understand the mechanism of tumor metastasis in the brain using in vitro BBB models. The patient glioblastoma spheroids have been planted into the microfluidic systems. It is efficient to investigate the drug-screening of high-tumor-killing capacity drugs through development of the BBB and patient glioblastoma spheroids models [119,120]. In Figure 4a, a glioblastoma (GBM)-on-chip model was established. A 3D bioprinting strategy was used to mimic the biochemical and biophysical properties of native GBM environment [115]. Moreover, recapitulation of the structural, biochemical, and biophysical properties of the native tumor microenvironment could be realized in-depth using the glioblastoma BBB-on-chip models.

### 5.2. Drug-Screening and Efficacy Evaluation

The drugs for treating brain diseases can be early screened based on the in vitro BBB models, including novel biopharmaceuticals and nanomedicines. A high-throughput BBB model has been used for initial drug permeability studies to identify molecules that can cross the BBB [81]. Permeability coefficients for model drugs (e.g., caffeine, cimetidine, and doxorubicin) were measured using the in vitro BBB system and showed good correlation with in vivo data. Bohye et al. developed an in vitro 3D BBB model with hydrogels for evaluating various brain-targeting drugs and drug carrier candidates [120]. The limitation of large biopharmaceuticals with low-efficient delivery into the brain has been investigated. Furthermore, the nanomaterials show attractive transfer ability as drug carriers through the BBB models, and they can prevent the degradation of drugs before delivery to the targeted area. Many stimuli-sensitive nanomaterials are designed, which can release the drugs under magnetic, heating, optical, and acoustic stimulation. Meanwhile, the 3D BBB models can be used for investigating nanoparticle transport mechanism. Ahn et al. designed a micro-physiological platform that recapitulates the key structure and function of the human BBB and enables 3D mapping of nanoparticle distributions in the vascular and perivascular regions. Their model precisely captures 3D nanoparticle distributions at cellular levels and demonstrates distinct cellular uptakes and BBB penetrations through receptor-mediated transcytosis [8].

### 5.3. Stem Cell-Based BBB Models in Personalized Medicine

Presently, the development of stem cell-based BBB models in personalized medicine has entered a new phase. Gad et al. created an entirely human BBB-Chip with induced pluripotent stem cell (iPSC)-derived brain microvascular endothelial-like cells (iBMECs), astrocytes, and neurons [69]. The iBMECs formed a tight monolayer that expressed markers specific to brain vasculature. The BBB-Chip exhibited physiologically relevant transendothelial electrical resistance and accurately predicted blood-to-brain permeability of various pharmaceuticals. Upon perfusing the vascular lumen with whole blood, the micro-engineered capillary wall protected neural cells from plasma-induced toxicity. Patient-derived iPSCs from individuals with neurological diseases predicted disease-specific lack of transporters and disruption of barrier integrity. By combining Organ-on-a-Chip technology and human iPSC-derived tissues, a neurovascular unit that recapitulates complex BBB functions has been created, providing a platform for modeling inheritable neurological disorders and advancing drug-screening as well as the development of personalized medicine.

### 5.4. Neurological Disorder Disease Modeling

The inflammatory response of neural disease lesion is from the aggregation and migration of the immune cells, the neutrophils, glial cells and astrocytes [121]. In neurological disorder diseases models, such as the AD models [122], the neuroinflammation is due to the activation of microglia and astrocytes. The inflammatory cytokines, such as tumor necrosis factor (TNF)-α and interleukin (IL)-1β, are released by the activated immune cells [123]. During the inflammatory response, cytokines and the immune cells participate in breaking the BBB barrier, which usually leads to the blood crush into the brain and causes an irreversible brain tissue impair [124]. Recently, a BBB-on-chip inflammatory model was developed to investigate the neutrophil infiltration in order to find properly therapeutic methods for neurological disorders [125]. The 3D BBB-on-chip model was developed to study neuroinflammation of the neurovascular disorders. Treated by TNF-α, the tight BBB could be disrupted along with the occurrence of ischemia. However, IL-8 (a potent neutrophil activator and a chemoattractant) treatment does not induce neutrophils to cross the BBB. Due to the good prevention of the infiltration of the neutrophils, this model can be further used as a desirable platform for developing new therapies for neurological disorders [24]. Bonakdar et al. built the reversible and irreversible electroporation model. They found the relationship between the distribution of affected cells and the degree of electroporation. In addition, different pulsed electric fields were used to investigate the perfusion rate of large molecules through the EC layers. This work showed that the drug transport across BBB could be regulated by the pulsed electric fields [126].

The BBB models for studying immune response may not have the same design criteria as the models for studying drug permeability [127]. There also exist in vitro BBB-related disease models for AD, PD, and other neurological disorders [128,129].

### 5.5. Neurobiology Research

The ability to control the microenvironment surrounding neuronal cells, such as crosstalk in cell–cell and cell–ECM integrations in the microfluidic platforms, can provide an in vivo-like niche for neural stem cells to differentiate into components of the nervous system. Jeon’s group has worked intensively to answer several neurobiological questions by providing appropriate experimental platforms that can resolve many limitations in conventional tissue culture [130]. Jeon’s microfluidics-based platforms have offered precise spatial temporal control of cellular microenvironments to explore various neuronal events. By combining microfluidics technology and the neurobiology, various technical problems in neurobiology can be overcome, such as culturing CNS neurons, isolating axons, patterning cultured neurons, controlling neurite outgrowth to mimic axonal injury and observing local protein synthesis in axons, axonal regeneration and axonal transport [131].

## 6. Conclusions and Perspective

The function of the BBB has attracted increasing attention in the field of neuroscience. There have been a lot of efficient in vitro BBB models used for investigations to treat various neurological diseases. In addition, micro- and nano-technologies are integrated with fabrication of in vitro BBB models, which provide the environment to better mimic the in vivo barrier structure. Microfluidic BBB models have become increasingly popular due to the ability to integrate the fluid flow, multicultural cell types, different design strategies, and the capability to measure TEER. Moreover, the choice of membrane materials, ECM hydrogels, or culture media, 2D or 3D systems all critically influence the in vitro BBB model behaviors. The microfluidic BBB models can provide a dynamic system mimicking blood flow, which is absent in the traditional static models. The shear stress due to the flow may help the formation of precise EC phenotype and reliable BBB integrity. In addition, surface modification of the devices can allow flexible design of the experiments [132]. Meanwhile, based on the microfluidics, many other platforms can be integrated with the in vitro BBB models for real-time monitoring of cell activities and providing electrical stimuli to the cells, etc. Apart from the well-designed methods, drug-screening can be very efficient with a high-throughput platform in microfluidic models. Many biomolecules and potential drugs have been evaluated in the microfluidic BBB models for their permeability. In addition, extracellular vesicles can be investigated as the alternative to biochemical drugs. These nanovesicles have good biocompatibility and can be easily transferred through the BBB. The in vitro BBB models have potential capabilities for other applications such as CNS disease modeling, personalized medicine development, brain tumor research, etc. Within less than a decade, the microfluidic BBB models have been rapidly developed and the next generation of organ-on-chip systems containing BBB structure (i.e., brain) is also possible.

## Figures and Tables

**Table 1:** Classification of the BBB models. hiPSC = human induced pluripotent stem cell, EC = endothelial cell, NSC = neuron stem cell.

Types of BBB Model	Culture System Conditions	Architecture for Culture	Limitations	Application	Ref.
static 3D model	multi-culture in transwell	Establish a coculture model by iPSCs derived neurons, astrocytes, pericytes to mimic in vivo neurovascular units	no shear stress	Confirmation of the relevant role of claudin subtypes for cellular tightness.	[53]
static 3D model	self-assembling multicellular BBB spheroids model	The spheroid core is comprised mainly of astrocytes, while brain endothelial cells and pericytes encase the surface, acting as a barrier that regulates transport of molecules	no shear stress and difficult to control the test	Screening and identifying BBB-penetrant cell-penetrating peptides.	[54]
static 2D model	polymer transwell membrane model	PLGA nanofiber mesh replace the traditional transwell membrane culture with hiPSC-EC and Astrocytes	no shear stress	A new, powerful tool for research on human BBB physiology and pathology higher TEER value and good barrier functions.	[55]
static 2D model	membrane free hydrogel BBB model	A collagen gel covered with a monolayer of brain microvascular endothelial cells	no shear stress and only ECs	Quantification of nanoparticle transcytosis and assessment of transendothelialdelivery of PEG-P(CL-g-TMC) polymersomes.	[56]
static 2D model	From mono- to transwell- to coculture BBB model	from the culture system with EC only, NSC only, EC and NSC transwell, to hECs/hNSC coculture	no shear stress with no pericytes and astrocytes	Assaying dynamic cellular interactions between hECs and NSCs and forming NVU.	[57]
static 2D model	Transwell model	Substituting pericytes with MSCs in fabricating BBB system	no shear stress and no astrocytes	Retaining the BBB phenotypes with TJ and permeability and up-regulating the pericytes mark.	[58]
static 2D model	Transwell model	iPSC-BMECs, astrocytes, pericyte, and neurons to form an isogenic human model	No fluidic flow and shear stress	Combining the BMECs, neurons, astrocytes, and brain pericyte-like cells from a single iPSC cell line to form an isogenic NVU model with optimal TEER.	[59]
Dynamic 3D spheroid model	microtiter plate	human primary astrocytes, human primary pericytes, hCMEC/D3	Difficult for integration test of BBB organoids	Developing a method for generation 90-multi-sized organoids reliably and reproducibly. Fabricating multi-sized BBB organoids and characterizing the drug dose response.	[60]
Dynamic 3D spheroid model	Hydrogel with glass dish	HUVECs, LM-4 cells, HL-60 cells	Complex fabrication method for large numbers of experiments	Establishing a new culture system in the lumen of glass culture dish. Observation of endothelial cells formation with different cell lines.	[61]
DIV-model	3D vasculogenic model	Human astrocyte and hECs	Too thick for the porous fiber	New platform for studying BBB.	[62]
DIV-model	QV-600 chamber multi-chamber perfusion system	PBMECs	Can only apply for the shear stress research	enhancing and maintaining TEER for longer.	[63]
microfluidics 2D model	sandwich design model	ECs and pericytes coculture with consistent fluid flow	low contact area between neuronal and vascular channels	Showing mechanical stimuli exerted by blood flow mediate both the permeability of the endothelial barrier and waste transport along the basement membrane.	[64]
microfluidics 3D model	3D vasculogenic hydrogel model	ECs coculture system with pericyte and astrocytes in collagen I gel	difficult to apply different shear stress	Build a new simple, cost-effective, and scalable in vitro platform for targeting neuroinflammatory conditions.	[65]

**Table 2:** **Examples of BBB-on-chip dynamic models.** hiPSC = human induced pluripotent stem cell, EC = endothelial cell, NSC = neuron stem cell, h = human, r = rat, m = mouse, UVEC = umbilical vein endothelial cords, BMEC = brain microvascular endothelial cell, iNPCs = induced neuron progenitor cells; PDMS = polydimethylsiloxane, PET = polyethylene terephthalate, PC = polycarbonate.

Culture Structure	Materials Used	Cell Type	Membrane	EC Layer Integrity Marker	TEER Value	Applications	Ref.
Vertical 2D culture	PDMS	hBMECs, pericytes, astrocytes, hiNPCs	PC	ZO-1	N/A	Provide a novel platform for modeling of BBB function and testing of drug toxicity and permeability regarding the CNS.	[78]
Tubular 3D culture	PDMS collagen gel	hMVECs, human astrocyte, human pericytes	N/A	ZO-1, VE-cadherin	40–50 Ω·cm^−2^	Astrocytes and pericytes coculture system enhances the integrity of BBB and provides better G-CSF and IL-6 secretion level than transwell.	[26]
Vertical chambers	PDMS	C6 astrocytes and bEnd.3 cells	PC	ZO-1	223–280 Ω·cm^−2^	Permeability of seven neuroactive drugs and TEER and predicting of BBB clearance of pharmaceuticals.	[79]
Parallel 3D chambers	PDMS	RBE4 cells and astrocytes	pores generated by lithography between two chambers	ZO-1	250 Ω·cm^−2^	Mimicking the in vivo microenvironment closely and showing better barrier properties.	[80]
Vertical 2D chambers	PDMS, 3D printed plastic, Ag/AgCl pellet electrode	iPSC-BMECs and astrocytes	0.4 µm PC	ZO-1, Claudin-5	4,000 Ω·cm^−2^	Evaluating the capacity of our microfluidic BBB model to be used for drug permeability studies using large molecules (FITC-dextrans) and model drugs.	[81]
Parallel 3D chambers	Organo Plate	hBMECs(TY10), human pericytes, human astrocytes	ECM gel	PECAM-1, Claudin-5, VE-Cadherin	N/A	Integrating a human BBB microfluidic model in a high-throughput plate-based format that can be used for drug-screening purposes.	[82]
Vertical 3D Chambers	PDMS	hBMECs, human astrocytes, human pericytes	8 µm PC	ZO-1, α-SMA	150 Ω·cm^−2^	Building an on-chip-BBB structure and function by cellular interactions, key gene expressions, low permeability, and 3D astrocytic network. Investigate the nanoparticles mechanism.	[8]
Layer-by-layer Sandwich coculture device	PMMA (Acrylic glass)	hBMECs, hUVEC, human pericytes	PET grids (laser cutting)	CD146, CD31	N/A	Constructing a dual channels microfluidic BBB model for high-resolution 3D localization microscopy of the cytoskeleton and 3D single-molecule-sensitive tracing of lipoprotein particles.	[83]
Vertical 2D Chambers	PDMS	hBMECs, human astrocytes, human pericytes	0.4 µm PET	ZO-1, Claudin-5, PECAM-1, GLUT-1, P-glycoprotein	17,000–27,000 Ω (hypoxia)/400–23,000 Ω (normaxia)	The hypoxia condition enhances the integrity of BBB model and this model provides a more precise model for drug-screening.	[77]
Parallel 3D multi-channels culture	PDMS	hUVEC, rat astrocytes in gel, rat neurons in gel	N/A	ZO-1, VE-cadherin	N/A	Inventing a new platform for the development of a more sophisticated and complex 3D in vitro neurovascular model and has good observation of neurons.	[84]
3D biomimetic vessel parallel microtubes	N/A	bEnd.3, U87 glioblastoma cells	porous microtube	ZO-1	71–75 Ω·cm^−2^	Fabricating a 1:1 scale biomimetic BBB model with satisfied TEER and capability for drug-screening.	[85]
2D vertical tandem multichambers	PDMS	hBMECs, human astrocytes, human pericytes	PC	VE-cadherin	N/A	The link system mimics the effects of intravascular administration of the psychoactive drug methamphetamine and determines the previously unknown metabolic coupling between the BBB and neurons.	[86]
3D vertical culture	n/A	bEnd.3 (murine ECs), N2a (murine brain neuroblastoma), C8-D1A (murine astrocytes), BV-2 (murine microglia)	Gel-cell matrix	claudin-5	N/A	Building a platform by measuring Organophosphate-based compounds (OPs) effects on barrier integrity, acetylcholinesterase (AChE) inhibition, viability and residual OP concentration with four model Ops.	[87]
3D vertical culture	PDMS, PC, Titanium elecrode	mBMECs, mouse astrocytes,	PC	ZO-1	3.6–4.5 kΩ (coculture)	Coculture system with multielectrodes integrated system and the enhance the TJ under shear stress.	[88]
3D 3 parrallel channels	PDMS, glass	hiPSC-ECs, human astrocytes, human pericytes	PDMS with 120 μm pores by fabrication	CD-31, F-actin	N/A	The microvascular model is fabricated by the vasculogenesis and provides transport of molecules.	[89]
3D 3 parrallel channels	PDMS, microhydrogel	hUVEC, Astrocytes	PDMS porous structure	CD-31, ZO-1	N/A	A NVU model was fabricated by perivascular network morphology and synaptic structures and test the permeability.	[74]
Vertical 2D channels	PDMS	hCMEC/D3 cell line or rEC, rat pericytes, rat astrocytes	0.45 PET	ZO-1, β-catenin	175 Ω·cm^−2^	The 2 or 3 cells coculture make it easy to observe the cell growth and primary cells show better BBB integration.	[90]
Vertical 2D channels	PDMS	RBE4 cell, rat neurons, rat pericytes, rat astrocytes	0.8 um PC	ZO-1	N/A	Isolation culture with the different chambers and test the neuroinflammation.	[91]
Vertical 2D channels	PDMS	rBMEC, rat astrocytes	collagen I gel	ZO-1, VE-cadherin	1300 Ω·cm^−2^	Replicating of the key structural, functional and mechanical properties of the blood–brain barrier. The interaction of cancer cells and astrocytes decrease the migration of the tumor.	[92]

## Data Availability

Not applicable.
